# Attitudes and concerns regarding booster dose of COVID-19 vaccine among Egyptian patients with autoimmune and rheumatic diseases: a cross-sectional survey study

**DOI:** 10.1186/s40545-023-00558-9

**Published:** 2023-04-05

**Authors:** Samar Tharwat, Elshimaa Saad Eleraky, Toqa Adel, Mohammed Kamal Nassar, Marwa Saleh

**Affiliations:** 1 Rheumatology & Immunology Unit, Department of Internal Medicine, Faculty of Medicine, Mansoura University, El Gomhouria St., Mansoura, 35511 Dakahlia Governorate Egypt; 2 Department of Internal Medicine, Faculty of Medicine, Horus University, New Damietta, Egypt; 3 Faculty of Medicine, Menoufia University, Shebeen El-Kom, Egypt; 4 Mansoura Nephrology and Dialysis Unit (MNDU), Department of Internal Medicine, Faculty of Medicine, Mansoura University, Mansoura, Egypt

**Keywords:** Booster dose, Vaccine, COVID-19, Egypt, Autoimmune, Rheumatic, Survey

## Abstract

**Background:**

COVID-19 booster dose vaccination acceptance and actual vaccination behavior is not known among Egyptian individuals with autoimmune and rheumatic diseases (ARDs). The aim of this study was to investigate the acceptability of booster dose of the COVID-19 vaccine, as well as the factors that drive and inhibit that acceptance among Egyptian patients diagnosed with ARDs.

**Methods:**

This interview-based, cross-sectional analytical study was carried out on ARD patients from 20 July to 20 November 2022. A questionnaire was created to assess sociodemographic and clinical data, as well as COVID-19 vaccination status and the intention to receive a COVID-19 vaccine booster dose, perception of health benefits of it in addition to the perceived barriers and/or concerns.

**Results:**

A total of 248 ARD patients were included, with a mean age of 39.8 years (SD = 13.2), and 92.3% were females. Among them, 53.6% were resistant to the COVID-19 booster dose, whereas 31.9% were acceptant and 14.5% were hesitant. Those who were administering corticosteroids and hydroxychloroquine shown significantly greater booster hesitancy and resistance (*p* = 0.010 and 0.004, respectively). The primary motivation for taking a booster dose among the acceptant group was own volition (92%). Most acceptants believed that booster dose can prevent serious infection (98.7%) and community spread (96.2%). Among the hesitant and resistant groups, the main concerns for booster dose were fear about its major adverse effects (57.4%) and long-term impact (45.6%).

**Conclusions:**

There is a low acceptability rate of booster dose of COVID-19 vaccine among Egyptian patients with ARD diseases. Public health workers and policymakers need to make sure that all ARD patients get clear messages about accepting the COVID-19 booster dose.

## Background

Coronavirus disease 2019 (COVID-19) is caused by the extremely contagious severe acute respiratory syndrome coronavirus 2 (SARS-CoV-2) [1].According to WHO reports, the burden of COVID-19 is exhibited in more than 652 million confirmed cases and 6.66 million fatalities worldwide as of December 23, 2022, with varying patterns of morbidity and mortality [2].COVID-19 pandemic has not yet ended, and mutant strains are still emerging [3]. This puts a lot of stress on health care facilities, the world economy, and society as a whole [4, 5].

Vaccination against SARS-CoV-2 is one of the most effective means of protecting communities against COVID-19 by preventing severe outcomes and mortality [6]. Since the start of COVID-19 mass vaccination efforts in December 2020, approximately 59.3% of the global population has been fully vaccinated [7]. Along with the increase in completely vaccinated persons toward herd immunity, a loss in humoral immunity after 6 months of vaccination with the second dose has been reported, leading to a new surge in COVID-19 infections [8, 9]. Recent research has shown that after the third and fourth doses of either the inactivated or mRNA vaccine, the occurrence of confirmed COVID-19 and severe disease drastically decreased [10].

Patients with ARDs who are receiving immunosuppressive therapy have been shown to have decreased humoral immune responses to routine SARS-CoV-2 vaccination regimens, putting them at a higher risk of severe COVID-19 and hospitalization [11, 12]. The United Kingdom's Joint Committee on Vaccination and Immunisation (JCVI) recommended on September 1, 2021, that severely immunocompromised individuals receive a third dose of COVID-19 vaccine to increase immunogenicity. In August, the United States (US) Food and Drug Administration (FDA) and US Centers for Disease Control and Prevention (CDC) made similar pronouncements [13]. During the early phases of the pandemic, there was a scarcity of data on the safety profiles of COVID-19 vaccinations in individuals diagnosed with ARDs. However, new evidence has shown that the benefits of vaccination in reducing the severe outcomes of COVID-19 in this high-risk patient group for severe COVID-19 outweigh the risk of potential vaccine-related adverse effects [14–16]. This conclusion was reached as a result of weighing the benefits of vaccination against the risk of potential vaccine-related adverse effects. According to findings published in 2021 by the COVID-19 Vaccination in Autoimmune Diseases (COVAD) study, the prevalence of COVID-19 vaccine hesitancy was 15%. The study also identified two major associated factors: a lack of data on the long-term safety of vaccines and a fear of vaccine-induced disease flares [17]. The amount of data on vaccine safety in ARDs and its impact on disease flares has increased, and the majority of it indicates that the vaccination has a favorable safety profile [14, 15, 18].

Therefore, repeated vaccinations may be particularly beneficial for individuals with ARDs and other susceptible populations [19]. It is postulated that a booster vaccination is necessary for patients with ARDs such as rheumatoid arthritis because it ensures the preservation of a high seroconversion rate and generates a twofold increase in the humoral response compared to the initial vaccination cycle [20].

Despite the early introduction of COVID-19 vaccines, vaccination hesitancy has arisen as a serious impediment to preventive measures [21, 22]. Additionally, booster dose vaccination acceptance and actual vaccination behavior may differ among Egyptian individuals with ARDs. As a result, understanding patients' perspectives on booster dosage vaccination is critical for both policymaking and service planning. Efforts are still required to overcome reluctance to take a booster dose [23]. So, it is crucial to comprehend the motivations and obstacles influencing acceptance of the booster vaccination among Egyptian patients with ARDs.

The aim of this study was to investigate the acceptability of a booster dose of the COVID-19 vaccine, as well as the factors that drive and inhibit that acceptance among Egyptian patients with ARDs.

## Methods

### Study design and settings

This interview-based, cross-sectional analytical study was conducted at the Rheumatology and Immunology Unit, Internal Medicine Department, Mansoura University Faculty of Medicine, Egypt, from 20 July to 20 November 2022.

### Study participants (inclusion and exclusion criteria)

Patients diagnosed with ARDs made up the target population. The following criteria were utilized in the selection process: (1) a minimum age of 18 years old, (2) a medical diagnosis of autoimmune and/or rheumatic disease and (3) a willingness to take part in the study. Patients who were diagnosed with cancer, who had serious organ damage, or who had severe neurological or mental health problems were excluded from the start.

### Sample size and sampling procedure

Patients who were diagnosed with ARDs were approached about taking part in the study. The data were collected using a sampling method known as convenience sampling. This calculation of the required sample size was carried out using G*Power; the result of interest is the acceptance and readiness of patients with ARDs to receive a booster vaccine against COVID-19. The results of a prior survey [24] that was carried out on the general population revealed that 78.3% of respondents were willing to receive booster doses. In light of this, with an effect size of 0.1, an alpha error of 0.05, and a power of study of 0.9, the sample size was determined to be 123 subjects.

### Questionnaire design

The questionnaire was designed by the authors after an exhaustive review of the literature [25, 26] and then translated into Arabic language. After that, the questionnaire was examined by five members of the rheumatology staff who had prior experience with questionnaire-based studies. The developed questionnaire underwent assessments for critical appraisal, content validity, and face validity. Four new items were added as a result, three were removed, and two were modified. Subsequently, it was pilot tested on 25 adult patients with ARDs of varying ages and backgrounds to evaluate its structure, clarity, and length, as well as the participants' general perception of the questionnaire, which resulted in a few minor modifications to the original questionnaire. Cronbach's alpha coefficient was used to determine the questionnaire's internal consistency [27]. The reliability coefficient was 0.85, indicating that the internal consistency was good. The data collected from participants who had taken part in the pilot study were excluded from the statistical analysis of the main study. The questions were created to be as straightforward and closed-ended as possible.

The participants were divided into three groups based on their response to the question of whether they intended to receive a booster dose of COVID-19 vaccination. The group that responded "Yes, definitely" or "Yes, possibly" was deemed acceptant. Those who responded "No, probably not" or "I don't know" were categorized as hesitant. When participants responded "No, definitely not" or "No, probably not", they were categorized as a resistant group.

There were 32 questions on the survey, which was made up of the following sections: the informed consent form, questions regarding sociodemographic data, clinical and therapeutic data, COVID-19 infection and vaccine-related information, intention to receive a booster dose of COVID-19 vaccine, perception of health benefits and booster dosage acceptance, and perceived barriers and/or concerns regarding the booster dose as the following.

Participants were informed that participation in the survey is voluntary and that they had the option to decline participation in the study. Their personal contact information and names will be kept strictly confidential, and only utilized for scientific research. By signing the consent form, they signified their willingness to participate in the study.

The first section included questions about sociodemographic data including gender, age, marital status, occupation, degree of education, smoking habit, family income, and socioeconomic status.

In the second section, clinical and therapeutic data were collected; participants were asked about the type of ARD they had, the duration of their disease, their self-reported health status (poor, fair, or good), any other associated comorbidities, and therapeutic data involving corticosteroids, conventional disease-modifying antirheumatic drugs (DMARDs), or biological agents.

Participants were asked if they had previously contracted COVID-19. They were also asked if they had a relative or friend who had been infected or died as a result of COVID-19 infection.

The participants were also inquired about their COVID-19 vaccination status. Those who received the COVID-19 vaccines were asked if they received it because they were convicted or because they were required to by state legislation, how many doses they received, and if they suffered any side effects from the vaccine.

Five questions evaluated how participants thought the health benefits of a booster dose of the COVID-19 vaccine, including questions about whether or not they believed it could protect against severe infection, prevent community spread, be as effective as the primary dose, and whether or not the benefits of the vaccine outweighed the risks.

Those who refused or were hesitant to receive the booster dose were asked about their perceived hurdles and/or concerns about taking the booster dose. Options were "Do not know how to register"; "too busy to receive the booster dose"; "booster dose is not available"; "vaccines do not work"; "fear of major adverse effects"; "fear of long-term effects"; and "uncertain about different brands".

### Questionnaire administration

The study was based on interviews. It was intended to take between 10 and 15 min to finish. The interviewer talked with as many ARD patients (inpatient or outpatient) as possible. A single interviewer performed face-to-face structured interviews with each participant. The interviews were conducted by all the researchers. This style of questioning makes it easier to investigate more complex subjects than self-administered types of questioning since the interviewer can provide more thorough explanations of the questions. The confidentiality and anonymity of the participants were ensured by not requesting any personal information.

### Ethical consideration

This study was conducted in compliance with the Helsinki Declaration's principles [28]. The Institutional Research Board of the Faculty of Medicine at Mansoura University approved the study protocol (approval registration number: R.22.07.1755.R1). All participants were provided with detailed information about the study, and their written informed consent was obtained.

### Statistical analysis

The responses given by the participants were recorded and entered in spread sheets created in excel. The data were analyzed using the Statistical Package for Social Science (SPSS) version 22 (IBM Corp., Armonk, NY). For quantitative data, parametric variables were presented as means with standard deviation (SD) and nonparametric variables as medians (min–max). Qualitative data were presented as numbers and percentages. The Shapiro–Wilk test was used to determine the normality of the variable distribution. For parametric variables, the one-way ANOVA test was used to compare the study groups, whereas the Kruskal–Wallis test was employed for nonparametric variables. To compare qualitative variables, the Chi-square test was utilized. A P value of less than 0.05 was deemed significant.

## Results

### Sociodemographic data and clinical characteristics

This study involved 248 Egyptian patients with ARDs (70.9% response rate); their mean age was 39.8 years (SD = 13.2). The majority of them were females (92.3%). Approximately two-thirds (68.5%) were married and 59.3% were from urban areas. Other sociodemographic data of the participants are shown in Table 1.

**Table 1:** Sociodemographic data of the study ARD patients according to their decision to receive a booster dose of COVID-19 vaccine (*n* = 248)

Variables mean ± SD or *n* (%)	Total (*n* = 248)	Booster dose Acceptant group (*n* = 79) 31.9%	Booster dose Hesitant group (*n* = 36) 14.5%	Booster Dose Resistant group (*n* = 133) 53.6%	*P*
Gender					
Male	19 (7.7)	3 (3.8)	6 (16.7)	10 (7.5)	0.055
Female	229 (92.3)	76 (96.2)	30 (83.3)	123 (92.5)	
Age	39.8 ± 13.2	45 ± 14.3	35.5 ± 13.12	37.9 ± 11.6	< 0.001*
Marital status					
Single Married Divorced Widow	55 (22.2) 170 (68.5) 9 (3.6) 14 (5.6)	14 (17.7) 55 (69.6) 3 (3.8) 7 (8.9)	13 (36.1) 22 (61.1) 0 1 (2.8)	28 (21.1) 93 (69.9) 6 (4.5) 6 (4.5)	0.228
Occupation					
Not employed Employed Retired Student	151 (60.9) 59 (23.8) 13 (5.2) 25 (10.1)	52 (65.8) 14 (17.7) 6 (7.6) 7 (8.9)	20 (55.6) 7 (19.4) 1 (2.8) 8 (22.2)	79 (59.4) 38 (28.6) 6 (4.5) 10 (7.5)	0.09
Residence					
Rural Urban	101 (40.7) 147 (59.3)	33 (41.8) 46 (58.2)	16 (44.4) 20 (55.6	52 (39.1) 81 (60.9)	0.824
Education					
Senior high school or below College degree or above	130 (52.4) 118 (47.6)	52 (65.8) 27 (34.2)	19 (52.8) 17 (47.2)	59 (44.4) 74 (55.6)	0.01*
Smoking habit					
Non_smoker Former smoker Current smoker	234 (94.4) 7 (2.8) 7 (2.8)	78 (98.7) 1 (1.3) 0	33 (91.7) 3 (8.3) 0	123 (92.5) 3 (2.3) 7 (5.3)	0.027*
Active lifestyle	125 (50.4)	30 (38.0)	19 (52.8)	76 (57.1)	0.025*
Family income					
Not enough Enough but no saving Enough and saving	126 (50.8) 94 (37.9) 28 (11.3)	43 (54.4) 25 (31.6) 11 (13.9)	18 (50) 14 (38.9) 4 (11.1)	65 (48.9) 55 (41.4) 13 (9.8)	0.68
Socioeconomic status					
Low Average High	105 (42.3) 139 (56) 4 (1.6)	39 (49.4) 38 (48.1) 2 (2.5)	13 (36.1) 23 (63.9) 0	53 (39.8) 78 (58.6) 2 (1.5)	0.42
Health insurance	61 (24.6)	13 (16.5)	11 (30.6)	37 (27.8)	0.119

* and bold values indiacte the significance of *p* < 0.05

**Fig. 1:**
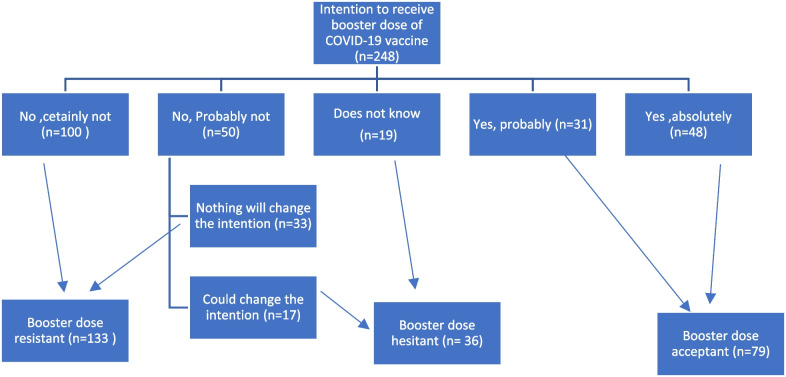
Classification of ARD patients according to their intention to receive booster dose of COVID-19 vaccine (*n* = 248)

The percentage of participants who were resistant to the booster dose of COVID-19 vaccination was high (53.6%), whereas only 31.9% were acceptant and 14.5% were hesitant as shown in Fig. 1.

As illustrated in Table 2, the most encountered ARDs were systemic lupus erythematosus (SLE) (36.3%), rheumatoid arthritis (RA) (27%), osteoarthritis (OA) (14.5%) followed by psoriatic arthritis (PsA) (2.8%), osteoporosis (2.4%) and systemic sclerosis (SSc) (2%). The median duration of the disease was five years. Hypertension (28.6%), chronic renal disease (11.3%), and diabetes mellitus (10.1%) were the most prevalent comorbidities. Those who received corticosteroids (*p* = 0.010) and hydroxychloroquine (*p* = 0.004) showed significantly greater booster hesitancy and resistance. In total, 110 (44.4%) participants reported prior infection with COVID-19, while 162 (65.3%) participants reported COVID-19 among their relatives and acquaintances. Approximately three-quarters (72.2%) received one or more doses of the COVID-19 vaccine. There was a statistically significant difference between the three groups in terms of COVID-19 vaccination (*p* < 0.001), since the vast majority of the acceptant group (98.7%) received at least one dose of the vaccine.

**Table 2:** Clinical and therapeutic data of the study ARD patients according to their decision to receive a booster dose of COVID-19 vaccine (*n* = 248)

Variables Median (min–max) or *n* (%)	Total (*n* = 248)	Booster dose acceptant group (*n* = 79)	Booster dose hesitant group (*n* = 36)	Booster dose resistant group (*n* = 133)	*P*
The rheumatic/autoimmune disease					
Systemic lupus erythematosus Rheumatoid arthritis Juvenile idiopathic arthritis Systemic sclerosis Myositis Vasculitis Sjögren's syndrome Mixed connective tissue disease Ankylosing spondylitis Psoriatic arthritis Gout Osteoarthritis Osteoporosis Fibromyalgia Behcet's disease Familial Mediterranean fever Others	90 (36.3) 67 (27.0) 1 (.4) 5 (2.0) 1 (.4) 4 (1.6) 4 (1.6) 3 (1.2) 4 (1.6) 7 (2.8) 3 (1.2) 36 (14.5) 6 (2.4) 5 (2.0) 3 (1.2) 2 (.8) 7 (2.8)	21 (26.6) 18 (22.8) 0 1 (1.3) 1 (1.3) 1 (1.3) 1 (1.3) 1 (1.3) 2 (2.5) 2 (2.5) 0 3 (3.8) 19 (24.1) 2 (2.5) 1 (1.3) 2 (2.5) 2 (2.5)	20 (55.6) 6 (16.7) 0 2 (5.6) 0 0 1 (2.8) 1 (2.8) 0 0 0 3 (8.3) 1 (2.8) 0 0 0 1 (2.8)	49 (36.8) 43 (32.3) 1 (.8) 2 (1.5) 0 3 (2.3) 2 (1.5) 1 (.8) 2 (1.5) 5 (3.8) 0 14 (10.5) 2 (1.5) 3 (2.3) 2 (1.5) 0 4 (3.0)	0.164
Disease duration (years)	5 (0.1–35)	5.5 (.10–32)	4 (.25–20)	5 (.10–35)	0.066
Self-reported health status					
Poor Fair Good	41 (16.5) 147 (59.3) 60 (24.2)	12 (15.2) 42 (53.2) 12 (15.2)	3 (8.3) 23 (63.9) 10 (27.8)	26 (19.5) 82 (61.7) 25 (18.8)	0.156
Associated comorbidities					
Hypertension Diabetes mellitus Chronic kidney disease Heart disease Lung disease	71 (28.6) 25 (10.1) 28 (11.3) 11 (4.4) 21 (8.5)	27 (34.2) 8 (10.1) 4 (5.1) 5 (6.3) 4 (5.1)	11 (30.6) 3 (8.3) 7 (19.4) 2 (5.6) 4 (11.1)	33 (24.8) 14 (10.5) 17 (12.8) 4 (3.0) 13 (9.8)	0.332 0.927 0.057 0.493 0.776
Therapeutic data					
Corticosteroids	141 (56.9)	34 (43.0)	24 (66.7)	83 (62.4)	0.010*
cDMARDs					
Methotrexate Hydroxychloroquine Mycophenolate mofetil Leflunomide	57 (23.0) 128 (51.6) 34 (13.7) 45 (18.1)	11 (13.9) 29 (36.7) 9 (11.4) 14 (17.7)	10 (27.8) 23 (63.9) 8 (22.2) 4 (11.1)	36 (27.1) 76 (57.1) 17 (12.8) 27 (20.3)	0.068 0.004* 0.264 0.444
Biologics					
TNF-alpha inhibitors Interleukin-6 inhibitors Interleukin-17 inhibitors JAK-inhibitors Rituximab	9 (3.6) 3 (1.2) 4 (1.6) 2 (.8) 7 (2.8)	0 1 (1.3) 1 (1.3) 1 (1.3) 2 (2.5)	1 (2.8) 0 0 0 1 (2.8)	8 (6.0) 2 (1.5) 3 (2.3) 1 (.8) 4 (3.0)	0.074 0.764 0.608 0.776 0.980
Prior COVID-19 infection	110 (44.4)	30 (38.0)	18 (50.0)	62 (46.6)	0.360
COVID-19 among relatives or friends	162 (65.3)	44 (55.7)	27 (75.0)	91 (68.4)	0.071
COVID-19 vaccination	179 (72.2)	78 (98.7)	25 (69.4)	76 (57.1)	< 0.001*

* and bold values indiacte the significance of *p* < 0.05; cDMARDs: conventional disease-modifying antirheumatic drugs

### COVID-19 vaccination data

Table 3 displays vaccine information for individuals who received at least one dose of the vaccine. The majority (57.5%) did so out of conviction. There was a statistically significant difference (*p* < 0.001) between the three groups on this point, as 82.1% of the acceptant group while 40% of the hesitant group, and 38.2% of the resistant group took it out of conviction. The majority of COVID-19 vaccination recipients (79.9%) received two doses, whereas just 12.3% received a booster dose. There was a statistically significant difference between the three groups in terms of the number of COVID-19 vaccination doses administered (*p* = 0.022).

**Table 3:** Vaccine data of the study ARD patients who received COVID-19 vaccine (*n* = 179)

Variables mean ± SD or *n* (%)	Total (*n* = 179)	Booster dose acceptant group (*n* = 78)	Booster dose hesitant group (*n* = 25)	Booster dose resistant group (*n* = 76)	*P*
Did you take the COVID-19 vaccine because you were convicted, or because you were forced to by state laws?					
I took it out of conviction I took it out of conviction and because of the laws I took it because of the imposed laws, not because I believe in it	103 (57.5) 32 (17.9) 44 (24.6)	64 (82.1) 9 (11.5) 5 (6.4)	10 (40.0) 9 (36.0) 6 (24.0)	29 (38.2) 14 (18.4) 33 (43.4)	< 0.001*
Received COVID-19 vaccine					
One dose Two doses Booster dose	14 (7.8) 143 (79.9) 22 (12.3)	2 (2.6) 64 (82.1) 12 (15.4)	1 (4) 19 (76) 5 (20)	11 (14.5) 60 (78.9) 5 (6.6)	0.022*
Brand of COVID-19 vaccine					
Pfizer Moderna Sinopharm Sinovac AstraZeneca Not sure	37 (20.7) 3 (1.7) 52 (29.1) 18 (10.1) 32 (17.9) 37 (20.7)	11 (14.1) 1 (1.3) 25 (32.1) 6 (7.7) 17 (21.8) 18 (23.1)	12 (48) 1 (4) 6 (24) 4 (16) 0 2 (8)	14 (18.4) 1 (1.3) 21 (27.6) 8 (10.5) 15 (19.7) 17 (22.4)	0.02*
Side effects experienced	135 (75.4)	53 (67.9)	20 (80)	62 (81.6)	0.123
Mild Moderate Severe	91 (50.8) 20 (11.2) 24 (13.4)	40 (51.3) 7 (9.0) 6 (7.7)	14 (56) 2 (8) 4 (16)	37 (48.7) 11 (14.5) 14 (18.4)	0.230
Reported side effects					
Headache Hyperthermia Pain at site of injection Muscle pain Weakness Spasm Nausea Rash Chills	65 (36.3) 104 (58.1) 112 (62.6) 80 (44.7) 38 (21.2) 5 (2.8) 17 (9.5) 4 (2.2) 24 (13.4)	24 (30.8) 42 (53.8) 49 (62.8) 25 (32.1) 9 (11.5) 3 (3.8) 4 (5.1) 0 3 (3.8)	10 (40) 13 (52) 18 (72) 15 (60) 10 (40) 0 7 (28) 2 (8) 7 (28)	31 (40.8) 49 (64.5) 45 (59.2) 40 (52.6) 19 (25.0) 2 (2.6) 6 (7.9) 2 (2.6) 14 (18.4)	0.398 0.328 0.517 0.009* 0.006* 0.593 0.003* 0.060 0.002*

* and bold values indiacte the significance of *p* < 0.05

Table 3 outlines the reported adverse reactions following vaccination. Those who experienced muscle pain, weakness, and chills were unwilling to receive a booster dose (*p* = 0.009, 0.006, and 0.002, respectively).

### Reasons for getting booster dose

**Fig. 2:**
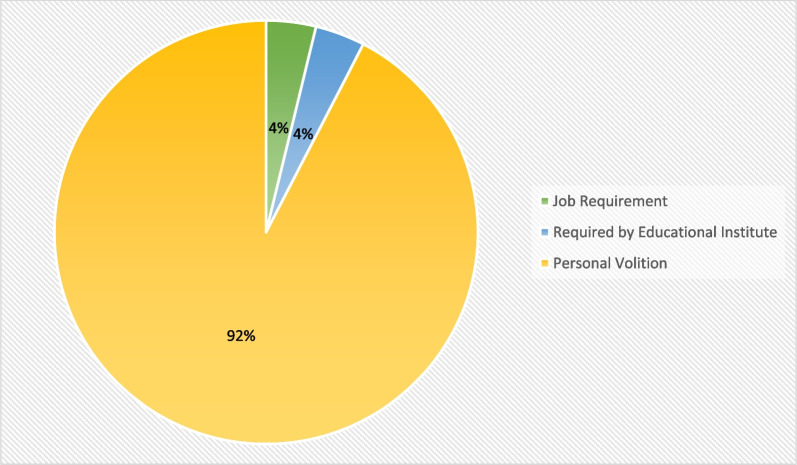
Reasons for getting booster dose in acceptant group (*n* = 79)

As illustrated in Fig. 2, the primary motivation for taking a booster dose among the acceptant group was own volition (92%).

### Perception of health benefits and booster dose

**Fig. 3:**
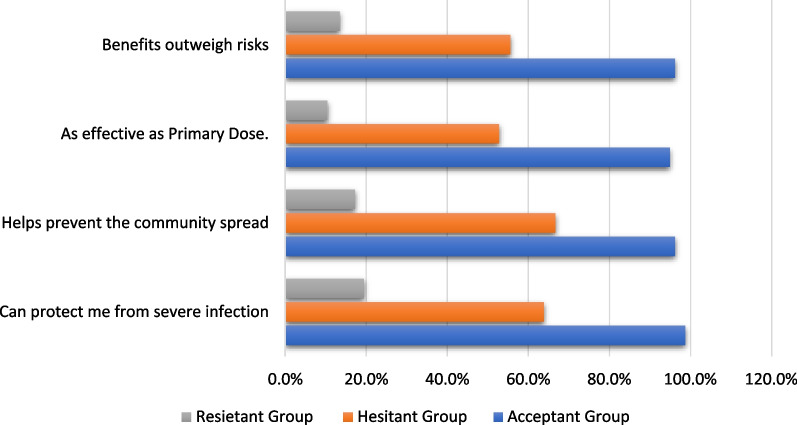
Perception of Health benefits and booster dose acceptance

There was a statistically significant difference between booster dosage acceptants and non-acceptants in terms of perception of health advantages and acceptance of booster dose. Most of the acceptant group feels that a booster dosage can guard against severe infection (98.7%) and prevent community spread (96.2%) as shown in Fig. 3.

### Perceived barriers and/or concerns for the booster dose

**Fig. 4:**
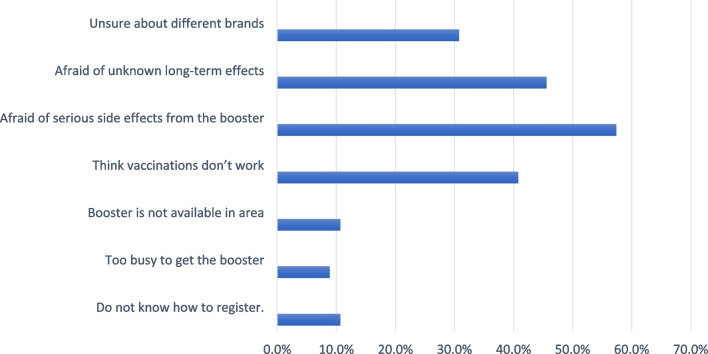
Perceived barriers and/or concerns for the booster dose in booster dose hesitant and resistant groups (*n* = 169)

Figure 4 depicts the barriers to booster dose acceptability in the hesitant and resistant groups. About 57.4% were worried about the major adverse effects of the COVID-19 vaccine, 45.6% were concerned about long-term impacts, and 40.8% believed vaccination did not work.

## Discussion

This survey was conducted among Egyptian patients with ARDs against the backdrop of ongoing COVID-19 outbreaks and the need to assess the acceptance rate of COVID-19 vaccine booster dose among these population and to understand why individuals were not accepting it. In this study, we used an interview-based survey to investigate ARD patients' perceptions of the benefits and drawbacks of a booster dose of COVID-19 vaccination, as well as their acceptance, hesitancy, and resistance. To the best of our knowledge, this is the first study of its kind among Egyptian patients with ARDs.

In this study, the percentage of participants who were resistant to the COVID-19 booster dose was high (53.6%), whereas only 31.9% were accepting and 14.5% were hesitant. In fact, the acceptance rate of booster dose varies greatly from study to study, ranging from 44.6% to 97.9%. In a meta-analysis of 14 studies involving 104,047 fully vaccinated people, 79.0% intended to accept a booster dose, 12.6% were unsure, and 14.3% intended to refuse [29]. Lack of trust and demographic traits were among the factors related with booster dosage hesitation. Additionally, demographic characteristics such as education level, marital status, regional disparities, and political affiliation were major contributors to booster dose hesitation among the general population [30].

The third (booster) dose is believed to be necessary for improved immunization [31]. In a study comparing breakthrough COVID-19 outcomes in booster-vaccinated patients with or without systemic rheumatic diseases, COVID-19-related hospitalizations were less likely in booster-vaccinated patients than in fully vaccinated or unvaccinated patients. While 4/60 (6.7%) unvaccinated persons died, neither the booster-vaccinated nor the fully vaccinated groups experienced mortality [32].

Due to the reduced vaccine efficacy in patients receiving systemic corticosteroids, it is advised that patients be vaccinated while their corticosteroid dose is at its lowest [33]. However, in the present study, those who received corticosteroids (*p* = 0.010) showed significantly greater booster hesitancy and resistance.

Our findings revealed that the majority (57.5%) of those who received at least one dose of the vaccine did so out of conviction. Individuals who were forced to obtain the initial vaccine doses by law, as well as those who received them as a result of both imposed legislation and conviction, showed more refusal/hesitancy to take the third booster dosage than those who received the initial doses as a result of conviction [34]. The main reason for refusing or delaying the vaccine booster dose is the conviction that there is adequate protection following primary vaccination and recovery from COVID-19 [35].

Only 12.3% of our study cohort received a COVID-19 booster dose. There is some early evidence that the booster dose is more effective in protecting against severe COVID-19 and lowering the risk of transmission. However, the social uncertainty about the safety and effectiveness of the vaccine may make people less likely to take the booster dose [36]. In general, the acceptability rate of the COVID-19 vaccine in Middle Eastern nations was observed to be relatively low. According to Abu-Farha et al., only 24.9% (*n* = 2925) of the population in several Middle Eastern nations accepted to be vaccinated against COVID-19 [37]. In addition, the rate of COVID-19 vaccine compliance in Arab countries was lower than the global rate [38, 39]. On the other hand, as of 10 July 2022, the cumulative uptake of the first booster COVID-19 vaccination in the European Union's total population was 52.9%. Booster dose vaccine uptake is higher (83.1%) among people aged 60 and older [40].

In our cohort, those who experienced muscle pain, weakness, and chills post COVID-19 vaccination were unwilling to receive a booster dose (*p* = 0.009, 0.006, and 0.002, respectively). The major reasons for refusing this booster dose of the COVID-19 vaccine could include negative past injection experiences [35]. It was found that adverse effects following booster vaccination are mild, and their frequency is the same as for the first or second dose. The most common adverse effects reported are injection site pain, tiredness, and myalgia (71.9%, 28.1%, and 21.8%, respectively) [41]. Most of these side effects are mild and happen because the immune system is responding to the booster dose [42, 43]

In this study, there was a statistically significant difference between booster dosage acceptants and non-acceptants in terms of perception of health advantages and acceptance of booster dose. According to previous studies [34, 44, 45], people are hesitant to get a booster vaccine because of concerns about the vaccine's safety, efficiency, and adverse effects; they also believe that the primary COVID-19 vaccination is sufficient; and they have a low perception of the disease's severity.

In fact, booster doses of COVID-19 vaccine are essential for patients with ARDs [29]. In patients with SLE and RA, the third BNT162b2 booster markedly enhanced humoral and cellular immunogenicity [46]. Additionally, recent investigations in healthy populations revealed that the third booster dose of mRNA vaccine improved protection against the Omicron variant, despite the fact that neutralizing antibody titres were lowered by sevenfold when compared to the ancestral variant [47–49].

Among the hesitant and resistant groups of our cohort, the main concerns for booster dose include fear about its major adverse effects and long-term impacts. In a cross-sectional study conducted in Jordan to examine how individuals feel about receiving a booster dose and the factors that influence their decision, nearly 45% of the respondents were willing to take the booster dose, and the most commonly mentioned reasons were the supposition of poor booster dose efficacy, followed by worries with the short time between the administration of the primary series and booster dose [34].

Indeed, we should reassure our patients that the vaccines currently in use are safe, have not been linked to underlying disease flares, and are far more enjoyable to receive than COVID-19 itself [50, 51].Previous studies [47, 52, 53] have shown that a booster dose of the currently available COVID-19 vaccinations reduces the risk and severity of omicron variant infection. To lower the risk of COVID-19 infection and illness severity, persons may consider obtaining a booster dose of the currently available COVID-19 vaccinations until variant-specific vaccines are available.

Scientists, researchers, and policymakers see a booster dose as a viable method of combating the COVID-19 virus; nevertheless, public acceptance of a booster dose is generally low due to a lack of trust and poor vaccine confidence among the general population [54–56].

To the best of our knowledge, this is the first study to investigate booster dose acceptability among Egyptian patients with ARDs. However, our study had some limitations that should be addressed. The study was cross-sectional and causal inferences could not be drawn and we are unable to provide information on alterations in booster dose perception over time. Furthermore, due to the dynamic nature of the COVID-19 pandemic, including the introduction of new SARS-CoV-2 viral variants, booster dosage acceptability among ARD patients may have changed. As a result, continuing monitoring of COVID-19 vaccination habits and attitudes in ARD patients is required.

## Conclusions

In conclusion, this survey gives useful information about COVID-19 vaccine booster hesitancy and the factors that may influence it in ARD patients. The acceptability of a booster dose was shown to be lower in Egyptian patients with ARDs compared to the general population. Concerns about vaccination safety, efficacy, and lack of confidence were identified as potential underlying causes of vaccine hesitancy. The offered observations and findings may be used as a basis for the development of future policies and public health measures intended to raise the COVID-19 booster dose vaccination rate. These results indicate the need for more intense acceptance campaigns that contribute to health promotion by leveraging health beliefs associated with booster acceptance, with a particular emphasis on people with ARDs.

## Data Availability

The datasets used and/or analyzed during the current study are available from the corresponding author on reasonable request.

## Abbreviations

ARDs: Autoimmune and rheumatic diseases
CDC: Centers for Disease Control and Prevention
COVID-19: Coronavirus disease 2019
DMARDs: Disease modifying antirheumatic drugs
FDA: Food and Drug Administration
OA: Osteoarthritis
PsA: Psoriatic arthritis
RA: Rheumatoid arthritis
SARS-CoV-2: Severe acute respiratory syndrome coronavirus 2
SLE: Systemic lupus erythematosus
SSc: Systemic sclerosis
US: United States
WHO: World Health Organization
